# Livestock farmer-reported knowledge and attitudes regarding agroforestry planning and management

**DOI:** 10.1007/s10457-024-01115-2

**Published:** 2025-01-15

**Authors:** Karolini Tenffen De-Sousa, Melanie Wright, Laura M. Cárdenas, Matheus Deniz, João Ricardo Dittrich, Maria José Hötzel, Daniel Enriquez-Hidalgo

**Affiliations:** 1 Programa de Pós-Graduação em Zootecnia, Universidade Federal do Paraná, Rua Dos Funcionários, 1540-Juvevê, Curitiba, Paraná 80035-050 Brazil; 2 Net Zero and Resilient Farming, Rothamsted Research, North Wyke, Okehampton, Devon EX20 2SB UK; 3 Grupo de Estudos em Bovinos Leiteiros, Faculdade de Medicina Veterinária e Zootecnia, Universidade Estadual Paulista, Botucatu, São Paulo, Brazil; 4 Laboratório de Etologia Aplicada e Bem-Estar Animal, Departamento de Zootecnia e Desenvolvimento Rural, Universidade Federal de Santa Catarina, Florianópolis, Santa Catarina Brazil; 5 Bristol Veterinary School, University of Bristol, North Somerset, Langford, Somerset BS40 5DU UK

**Keywords:** Expertise, Farm owner, Integrated systems, Opinion, Silvopasture

## Abstract

This study aimed to explore the knowledge and attitudes of livestock farmers from the United Kingdom regarding agroforestry planning and management issues. The farmers (n = 48) answered an online survey with demographic, open, closed and Likert scale questions. Almost half of the participants said they need more information to successfully plan and manage an agroforestry system, and self-reported low knowledge on management practices related to trees. Participants stated they did not expect to receive technical support from governmental agencies to maintain the agroforestry area. However, they would like to improve their knowledge through field days, courses, and Internet sources. Benefits to the environment, animals and farm profitability were considered central to successful agroforestry systems. In conclusion, participants cannot successfully plan and manage agroforestry, but they are willing to improve their knowledge and skills.

**Supplementary Information:**

The online version contains supplementary material available at 10.1007/s10457-024-01115-2.

## Introduction

Agroforestry systems present a duality as they provide substantial environmental benefits and enhance farm resilience to climate change, yet their adoption remains low. Among the benefits, agroforestry systems can help livestock sector to achieving Net Zero Carbon target, further to biodiversity increase, and providing livestock shade and shelter. Due to this range of benefits, the United Kingdom (UK) government offers financial support for tree introduction on farmlands (Rural Payments Agency 2021) and for farmers with an existing agroforestry system on their land (Mosquera-Losada et al. 2018). Regardless of the financial support from the government, one question that should be addressed in future policy is “Are the farmers prepared to plan and manage an agroforestry system?”.

Achieving the silvopastoral system’s potential benefits for the environment, animals, and landscape requires proper planning, design and maintenance (Jose et al. 2019). This includes the choice of tree species and design of the trees (Jose et al. 2019). Management practices like periodic pruning and thinning are important to allow more light input to improve and sustain the growth of trees and pastures (Brunetti et al. 2022; Pezzopane et al. 2020). When an agroforestry system is introduced at a farm, the farm system becomes more complex since it is necessary to manage tree-pasture-animal synchronically. The complexity of the agroforestry system demands technical knowledge that most farmers may not have or need to improve. Therefore, specialised technical support is needed for this system to be widely adopted and to maximise its benefits. Offering technical support to farmers can increase the chances of success in the implementation of agroforestry systems. This study aimed to explore the knowledge and attitudes of UK livestock farmers to the implementation of agroforestry in their farms, as well as any management issues.

## Material and methods

This study was approved by the Faculty of Health Science Research Ethics Committee (FREC) of the University of Bristol, protocol number 4.219.938/ 2020.

This study was exploratory in nature and based on a participant convenience sample. Livestock farmers from the UK were invited to participate in an online survey from January to July 2022 via the Google Forms platform. Participants were invited exclusively online, with the link to access the survey sent to e-mail lists of different organizations and published on social media platforms such as LinkedIn and Facebook, targeting livestock farmers. Additionally, each participant was encouraged to further promote the survey (snowball sampling method; Goodman 1961). Conditions to participate in this survey were that the participant was at least 18 years old, had income from animal production, and was available and interested in voluntarily answering a questionnaire covering the general theme of “Knowledge and attitudes of livestock farmers regarding activities associated with agroforestry planning and management". Only participants who agreed with the consent form were directed to the survey. The questionnaire included one open-ended, 14 multiple-choice, and three 5-point Likert scale questions divided into two sections (see supplementary file). Section 1 focused on demographic data, and Sect. 2 covered the participants’ knowledge and attitudes of toward information to plan (tree species selection, tree spacing, tolerance of pasture to shade and how to protect trees in the early years) and manage an agroforestry system [preventing damage from animals, thinning, pruning, replanting trees, maintenance of protective fences, grassland management (grazing management, livestock stocking rate and rotational grazing), soil fertility amendments].

From a total of 61 responses, 13 were eliminated because participants did not have animal production income, resulting in a final sample of 48 completed questionnaires. We used a qualitative approach to generate data to interpret and understand the knowledge of participants (Guest et al. 2012). To identify if there was a difference in the participants' knowledge the data from 5-point Likert scale were submitted to confirmatory analysis by the Kruskal–Wallis test. When significant, multiple comparisons were performed by the Bonferroni test, at the confidence level of 95%. All analyses [descriptive and confirmatory] were performed in R using the software RStudio (R Core Team 2023).

## Results

Participants were mainly male (83%) and 50% were older than age 50. Fifty-four percent of participants had more than 21 years of farming and 33% had a postgraduate degree (details of participants’ profile are shown in Table S1). Seventy-three percent had an agroforestry system at their farm; of these 57% said that agroforestry represents 5% of farm area; 37%, that it represents 6 to 25%, and 5.6% more than 26% of farm area.

**Fig. 1:**
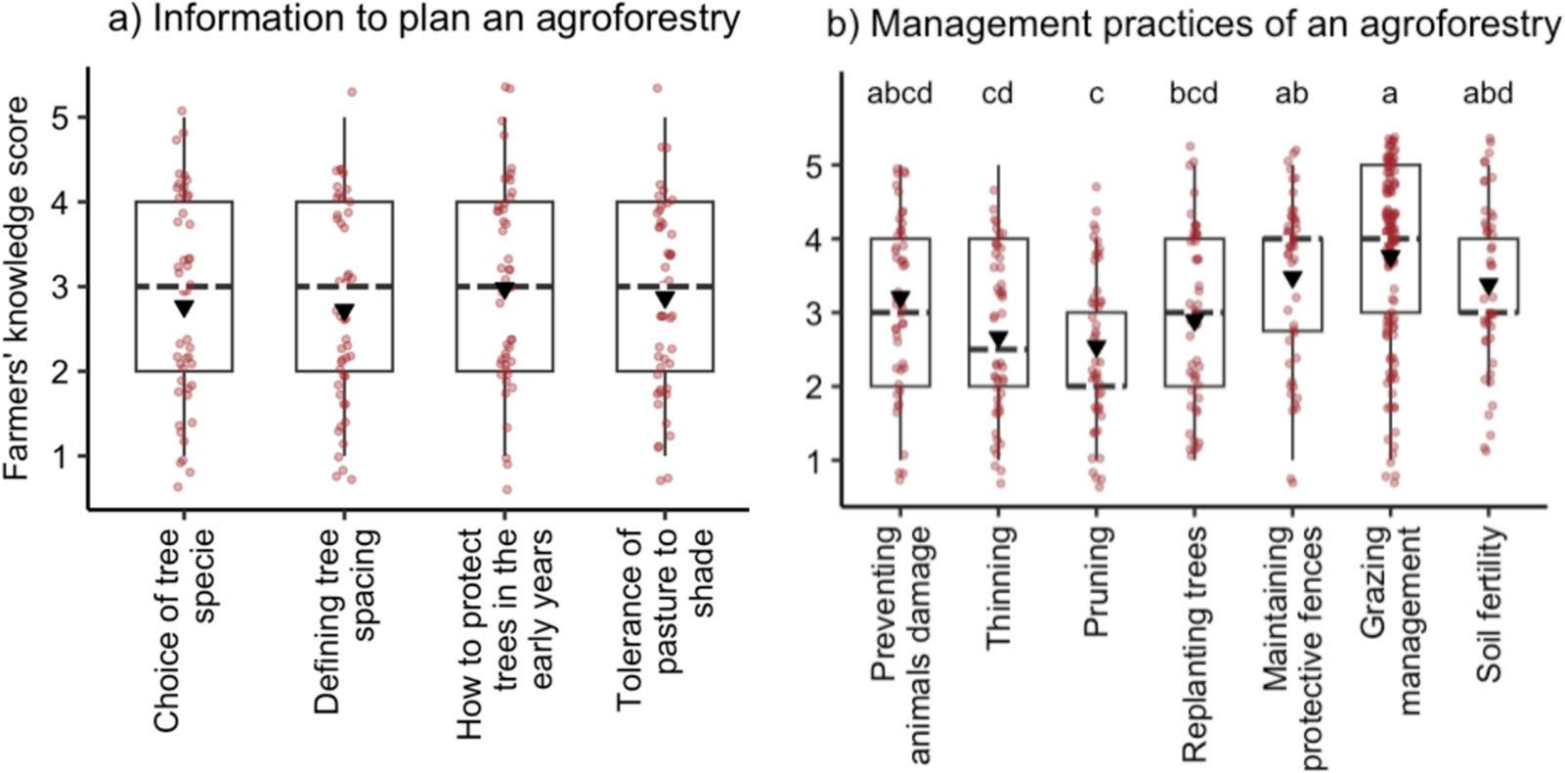
Livestock farmers’ (n = 48) knowledge score (Likert scale 1–5). Scores ≤ 3 indicate basic knowledge, a score of 3 indicates a neutral knowledge, and scores ≥ 3 indicate advanced knowledge. The black triangle in the boxplot represents the mean, the black dashed line represents the median, and the limits of the boxes represent the 25th and 75th percentiles (the first and third quartiles). The red points were jittered by ‘geom_jitter’ function (ggplot2 package; Wickham 2016)

Fifty-five percent of participants said that they did not have enough information to successfully plan and manage an agroforestry system. However, 62% would like to improve their skills in agroforestry management practices. There was no difference (*p* = 0.77, Fig. 1a) on participants’ knowledge score regarding the information to plan an agroforestry system. However, there was difference in participants’ knowledge scores related to the practices to manage the agroforestry system (*p* < 0.05; Fig. 1b).

Seventy-one percent of the participants believed that having technical support is important for the successful planning of agroforestry. However, 74% did not expect to receive technical support from governmental agencies to maintain the agroforestry area. Most participants (83%) said that when they searched for agroforestry technical information, the main sources of information were: the internet and research centers (both 31%), followed by online forums (17%), community groups (17%), social media (11%, including TikTok, Facebook, Twitter, or Instagram), books (9%) and universities (6%). Further, to support their own research, participants would like to receive technical support through participation in field days (74%), courses/trainings (45%), online forums (43%), printed materials (31%), and regular advisor visits (20%).

**Fig. 2:**
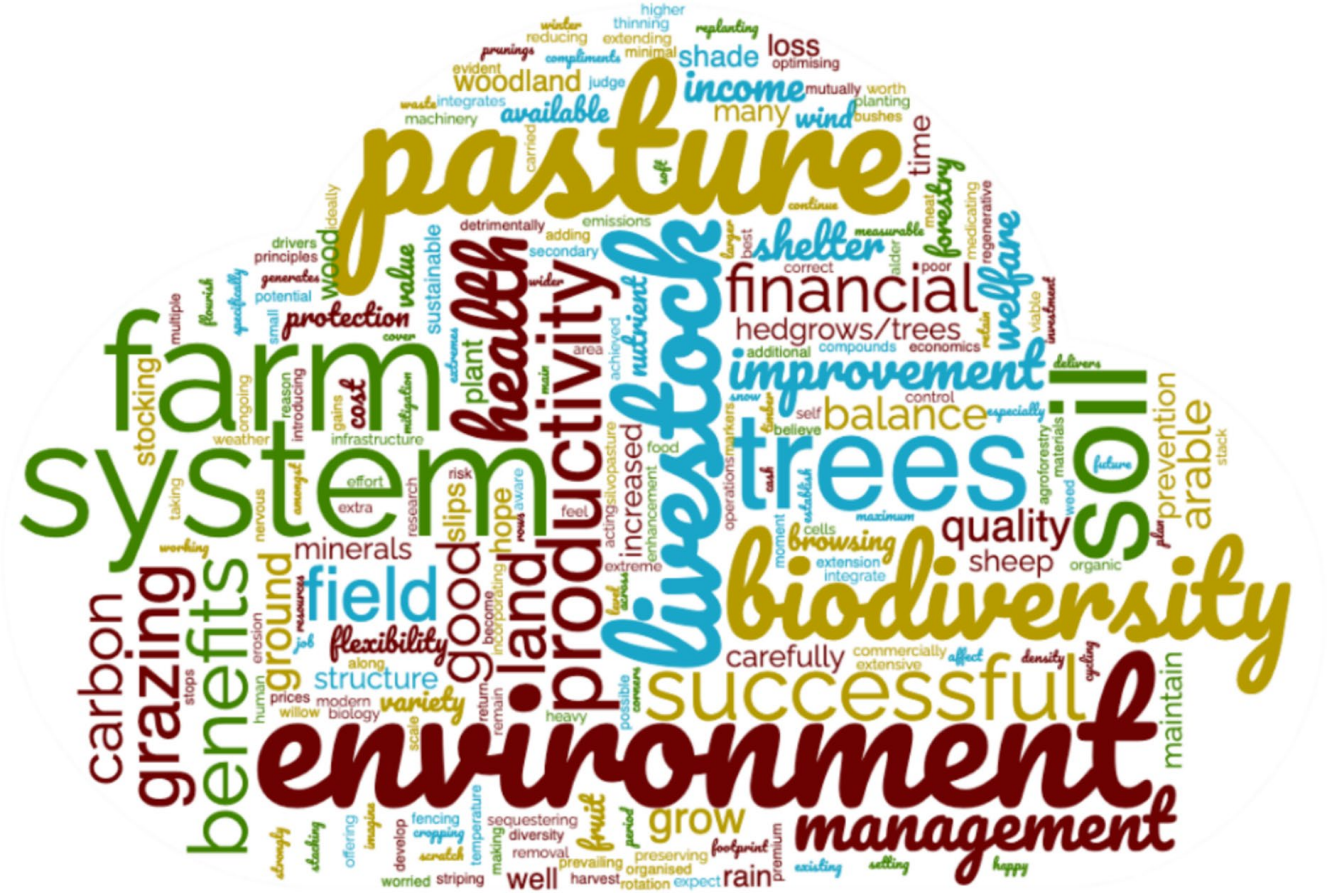
Word clouds generated using the most frequently used words on the response (n = 48) to the question “In your opinion, what does a successful agroforestry system mean?” The words that appear in larger font were used more frequently

To capture the participants’ main opinions on the open-ended questions about what a successful agroforestry system means, we used a word cloud (Fig. 2). Fifty-six percent of the participants mentioned financial aspects (“Successfully balancing farming with the environment. If we can do this and maintain a good level of productivity, we feel this is beneficial and worth our extra time, effort and investment. We believe strongly we should not be taking more than we give to the land.”—Participant 40; “More productive overall than just pasture, with higher welfare and premium prices for the meat. Co-benefits from cash cropping the trees.”—Participant 23), while 42% mentioned benefits to the environment and 39% indicated benefits to the animals (“Low cost, high welfare, high nature-value, low carbon footprint”- Participant 33; “Mutually beneficial systems. Shade for cattle, willow for minerals etc., prevention of soil erosion to river, improved nutrient cycling in these areas through varying root structures. Minimal loss of productive grazing land, machinery operations should be able to continue where possible.”—Participant 59).

## Discussion

Overall, UK livestock farmers participating in this study did not have enough knowledge to plan and manage an agroforestry system. Additionally, they demonstrated more awareness of grassland management than practices related to the management of trees, which is unsurprising, as effective pasture management is the most affordable feed source for livestock. However, in a complex system like agroforestry, if pastures and trees are not managed simultaneously, neither will perform optimally. The involvement of foresters could strengthen the synergy between trees and pastures, yielding significant benefits for food supply (Wilkens et al. 2021).

Despite our farmers’ positive self-perceived awareness of grassland management (rotational grazing, livestock stoking rate and grazing management), the lack of adequate knowledge exchange with farmers has been pointed out as one of the main problems in improving farmers’ skills in grassland management (Van Den Pol-van Dasselaar et al., 2020). Most participants did not expect to receive government assistance, but they are willing to improve their knowledge of agroforestry systems, through field days, courses, and Internet sources. Collaborative efforts between farmers and agricultural advisors in a Wisconsin, USA community have successfully promoted the growth of silvopasture systems in the region, demonstrating the impact of community-driven knowledge exchange and support (Mayerfield et al., 2023).

Aligning agroforestry with financial returns was one of the desires of participants in this study. Noteworthy, previous studies in the UK reported the perception of low financial return from agroforestry as a barrier to the adoption of agroforestry (Lawrence and Dandy 2014; Felton et al. 2023). Farmers will adopt practices if they are beneficial in their context, if they have the necessary skills to implement the practice successfully, and if they are fully aware of the practice and its benefits and costs. Marais et al. (2019) suggest the key to motivate farmers adoption is striking an appropriate balance between practicality and the relevance of outputs to decision-making. This may indicate that more than financial support from the government to introduce trees on farms is needed to spread agroforestry if this policy is not integrated into others, as forestry and agriculture are still seen as separate entities in UK policy and funding schemes are unclear (Venn and Burbi 2023). Farmers can be the most effective spokespersons for agroforestry practices if they know, from personal experience, that by introducing trees on their farms, they can improve profitability and quality of life.

Despite most of the participants in this study having an agroforestry area on their farm, many reported only possessing a minimal understanding of tree management practices, such as thinning and pruning. This may indicate that farmers carried out few or no management practices with trees. Taking care of the tree canopy is important to optimize light exposure to improve the growth of trees and pasture (Jose et al. 2019). According to Felton et al. (2023), the lack of knowledge of tree management is no longer a significant barrier to agroforestry adoption as English farmers have become more aware of these practices. In our study, most farmers were willing to improve their knowledge of agroforestry systems through participation in field days. This demonstrates that farmers may prefer advice in the form of practical examples, which is related to their reliance on learning from their peers (Rust et al. 2022). A more practical and less theoretical education has been pointed out as a promising strategy for advisor training (Landini et al. 2017); afterwards, advisors can use this same approach in farmer training. Our findings, combined with previous studies, points toward a dissemination of agroforestry based on practical examples like model farms and events open to producers.

## Conclusion

We found that farmers have low levels of knowledge on aspects related to the planning and management of an agroforestry system, but they are willing to improve their skills without expectation of receiving government assistance. Farmers are more aware of routine practices such as grassland management than the management of trees. The farmers’ motivation to improve their skills in agroforestry systems should be combined with on-farm practical examples to make the UK Net Zero Carbon policy more effective.

The results of our study can help researchers and advisors to align their efforts with the expectations of the farmers. Furthermore, to ensure that government incentives are effectively utilized, it is important to establish clear communication and a common language among all stakeholders, including farmers, advisors, and government officials.

## Supplementary Information

Below is the link to the electronic supplementary material.

Supplementary file1 (DOCX 30 KB)

## Data Availability

The datasets generated for this study are available on request to the corresponding authors.
