# Supplementary material for: Livestock farmer-reported knowledge and attitudes regarding agroforestry planning and management
Source: Agrofor Syst. 2025 Jan 15;99(1):28. doi: 10.1007/s10457-024-01115-2 (PMC11735556; doi:10.1007/s10457-024-01115-2)
Supplement: Supplementary file 1 — Supplementary file1 (DOCX 30 KB) [file 10457_2024_1115_MOESM1_ESM.docx]

**Livestock farmer-reported knowledge and attitudes regarding agroforestry planning and management**

Karolini Tenffen De-Sousa, Melanie Writght, Laura Cárdenas, Matheus Deniz, João Ricardo Dittrich, Maria José Hötzel, Daniel Enriquez-Hidalgo

**Supplementary File**

**Table S1.** Profile of the 48 livestock farmers from United Kingdom that participated in the survey on knowledge and attitude regarding agroforestry planning and management.

| Demographics | Variables | n | % |
| --- | --- | --- | --- |
| Age (years) | 18 – 29 | 5 | 10% |
|  | 30 - 39 | 7 | 15% |
|  | 40 – 49 | 12 | 25% |
|  | 50 - 59 | 12 | 25% |
|  | 60 or more | 12 | 25% |
| Gender | Female | 8 | 17% |
|  | Male | 40 | 83% |
| Education | Elementary school | 2 | 4% |
|  | College | 12 | 25% |
|  | Technical degree | 6 | 12% |
|  | Bachelor’s degree | 12 | 25% |
|  | Post-graduate degree | 16 | 34% |
| Years farming | Up to 10 | 11 | 23% |
|  | 11 - 20 | 11 | 23% |
|  | 21 or more | 26 | 54% |
| Total farm area (ha) | < 10 | 5 | 10% |
|  | 11 - 100 | 15 | 31% |
|  | > 101 | 28 | 59% |
| Farm animals | Cattle - Beef | 36 | 75% |
|  | Cattle - Dairy | 13 | 27% |
|  | Sheep | 26 | 54% |
|  | Poultry | 7 | 14% |
|  | Pigs | 2 | 4% |

**Questionnaire**

**Section 1 -** socio-demographic questions

- What is your gender?
- Male
- Female
- Other
- What is your age (years)?
- 18 – 29
- 30 – 39
- 40 – 49
- 50 - 59
- More than 60
- What is your highest level of formal education? (Tick one box)
- Elementary school
- College
- Technical degree
- Bachelor’s degree
- Graduate degree
- Post-graduate degree
- Other (Please describe)
- How many years have you been in farming?
- 0 to 5 years
- 6 to 10 years
- 11 to 15 years
- 16 to 20 years
- More than 20 years
- What is the size of your farm in hectares?
- I have no farm (tenants/ licensees)
- Less than 5
- 5 to 10
- 11 to 30
- 31 to 100
- 102 to 200
- More than 200
- What type of farming operation(s) do you run? Please tick all the boxes that apply to you.
- Cattle – dairy
- Cattle – beef
- Sheep
- Pigs
- Poultry
- Other (please specify)
- Agroforestry is where trees are deliberately combined with agriculture and/or livestock on the same piece of land. Do you have any agroforestry area on your farm (examples: silvoarable, silvopastoral, riparian forest buffers, windbreak/shelterbelts, forest farming, orchards and hedgerows)?
- Yes (Go to section 4 – Farm area of agroforestry system)
- No (Go to section 6 – Farmers’ without na agroforestry system)
- Do you have income from any type of animal production?
- Yes (**Go to next section)**
- No (**the questionnaire ENDs here)**
- What is the percentage of your farm area devoted to the agroforestry system? (Question only for farmers that stated to have as agroforestry area)
- Less than 5%
- 6 to 25%
- 26 to 50%
- 51 to 75%
- More than 76%

________________________________________________________________

**Section 2 -** Knowledge and attitudes of participants toward information to plan and manage an agroforestry system

- In your opinion, what does a successful agroforestry system mean? How would you hope to achieve that success? (**open question**)
- Do you have expectations of getting technical support (e.g. courses, training, field days and regulars visits) from the governmental agencies in maintaining agroforestry?
- Yes
- No
- How would you like to get technical support from the government in maintaining agroforestry? Please tick all the boxes which would you like.
- Courses
- Training
- Field days
- Regular visits
- Online forum
- Printed material (e.g.: guidebook)
- Select the most important sources from where you get your agroforestry technical information nowaday.
- Internet (e.g. google)
- Neighbours
- Social media (e.g. Tiktok, Facebook, Twitter, Instagram)
- Community groups
- Universities
- Research centres
- Books
- Online forum
- I do not search agroforestry technical information

**SECTION – All livestock farmers**

- How much do you agree/ disagree with the following sentences:

| **Sentences** | Strongly disagree | Disagree | Neutral | Agree | Strongly agree |
| --- | --- | --- | --- | --- | --- |
| “I think that the technical support is important for planning agroforestry.” |  |  |  |  |  |
| “I feel that I have all the information necessary to successfully introduce agroforestry.” |  |  |  |  |  |
| “I feel that I have all the information necessary to successfully manage my agroforestry.” |  |  |  |  |  |
| “I would like to engage in courses to improve my knowledge on the management of agroforestry.” |  |  |  |  |  |

| **Information to implement** | - How do you rate your level of knowledge about this information to implement and manage agroforestry? | | | | |
| --- | --- | --- | --- | --- | --- |
|  | Below basic | Basic | Neutral | Proficient | Advanced |
| Tree species selection |  |  |  |  |  |
| Tree spacing |  |  |  |  |  |
| Tolerance of pasture to shade |  |  |  |  |  |
| How to protect trees in the early years |  |  |  |  |  |
| **Practical management** | Below basic | Basic | Neutral | Proficient | Advanced |
| Thinning |  |  |  |  |  |
| Pruning |  |  |  |  |  |
| Replanting trees |  |  |  |  |  |
| How to prevent damage from animals to trees |  |  |  |  |  |
| Maintenance of protective fences |  |  |  |  |  |
| Grazing management |  |  |  |  |  |
| Soil fertility amendments |  |  |  |  |  |
| Livestock stocking rate |  |  |  |  |  |
| Rotational grazing |  |  |  |  |  |

**(the questionnaire ENDs here)**
